# Identification of Exosome-Associated Biomarkers in Diabetic Foot Ulcers: A Bioinformatics Analysis and Experimental Validation

**DOI:** 10.3390/biomedicines13071687

**Published:** 2025-07-10

**Authors:** Tianbo Li, Lei Gao, Jiangning Wang

**Affiliations:** Department of Orthopedics Surgery, Beijing Shijitan Hospital, Capital Medical University, Beijing 100038, China; litianbo3285@bjsjth.cn (T.L.); gaolei3337@bjsjth.cn (L.G.)

**Keywords:** diabetic foot ulcers, exosome-associated biomarkers, machine learning, immune infiltration, molecular docking

## Abstract

**Background:** Diabetic foot ulcers (DFUs) are a severe complication of diabetes and are characterized by impaired wound healing and a high amputation risk. Exosomes—which are nanovesicles carrying proteins, RNAs, and lipids—mediate intercellular communication in wound microenvironments, yet their biomarker potential in DFUs remains underexplored. **Methods:** We analyzed transcriptomic data from GSE134431 (13 DFU vs. 8 controls) as a training set and validated findings in GSE80178 (6 DFU vs. 3 controls). A sum of 7901 differentially expressed genes (DEGs) of DFUs were detected and intersected with 125 literature-curated exosome-related genes (ERGs) to yield 51 candidates. This was followed by GO/KEGG analyses and a PPI network construction. Support vector machine–recursive feature elimination (SVM-RFE) and the Boruta random forest algorithm distilled five biomarkers (DIS3L, EXOSC7, SDC1, STX11, SYT17). Expression trends were confirmed in both datasets. Analyses included nomogram construction, functional and correlation analyses, immune infiltration, GSEA, gene co-expression and regulatory network construction, drug prediction, molecular docking, and RT-qPCR validation in clinical samples. **Results:** A nomogram combining these markers achieved an acceptable calibration (Hosmer–Lemeshow *p* = 0.0718, MAE = 0.044). Immune cell infiltration (CIBERSORT) revealed associations between biomarker levels and NK cell and neutrophil subsets. Gene set enrichment analysis (GSEA) implicated IL-17 signaling, proteasome function, and microbial infection pathways. A GeneMANIA network highlighted RNA processing and vesicle trafficking. Transcription factor and miRNA predictions uncovered regulatory circuits, and DGIdb-driven drug repurposing followed by molecular docking identified Indatuximab ravtansine and heparin as high-affinity SDC1 binders. Finally, RT-qPCR validation in clinical DFU tissues (*n* = 5) recapitulated the bioinformatic expression patterns. **Conclusions:** We present five exosome-associated genes as novel DFU biomarkers with diagnostic potential and mechanistic links to immune modulation and vesicular transport. These findings lay the groundwork for exosome-based diagnostics and therapeutic targeting in DFU management.

## 1. Introduction

The prevalence of diabetes mellitus (DM) is rising dramatically worldwide, posing significant challenges to global healthcare systems [1]. Diabetic foot ulcers (DFUs) represent one of the most devastating and costly complications of diabetes and are characterized by impaired wound healing and a high risk of infection [2]. Throughout their lifetime, DM patients face a 15% risk of developing DFUs [3], and alarmingly, approximately 36.55% of these patients ultimately require amputation surgery [4]. The post-amputation five-year mortality rate ranges from 39% to 68% [5], highlighting the exceptionally poor survival outcomes associated with this complication. Consequently, DFUs have emerged as one of the leading causes of disability and mortality among diabetes patients [6].

Wound healing is a precisely orchestrated physiological process aimed at restoring anatomical integrity with similar functionality [7]. In diabetic patients, however, this process is significantly compromised due to impaired glucose metabolism, peripheral vascular and nerve lesions, and multiple other factors that collectively contribute to delayed wound healing [8]. Current conventional DFU treatments include the local debridement of necrotic tissue, vascular reconstruction, infection control, and advanced dressing applications. Despite these interventions, the disability and mortality rates associated with DFU have not significantly improved. Traditional therapeutic approaches fail to address the fundamental pathological mechanisms of microcirculation occlusion, neurological damage, and tissue impairment [9], indicating an urgent need for novel therapeutic strategies based on a deeper understanding of DFU pathophysiology.

Recent advancements in high-throughput sequencing and bioinformatics have significantly enhanced our understanding of diseases at the molecular level, facilitating the discovery of disease-related risk genes with greater precision and efficiency [10]. These technologies offer promising avenues for revolutionary advances in disease diagnosis and treatment. Therefore, there is a compelling need to explore differentially expressed genes (DEGs) in DFUs to provide more precise and optimized treatment approaches, ultimately reducing the disability and mortality rates for patients with this devastating complication.

Over the past decade, exosomes have garnered significant scientific attention for their potential role in DFU pathology and treatment [11]. Exosomes are a type of extracellular vesicles (EVs) secreted by almost all cell types and are characterized by a double-layer membrane structure and particle sizes ranging from 30 to 150 nm [12]. These nano-sized vesicles carry diverse cargo, including RNA, DNA, proteins, and other bioactive molecules [13], functioning as important mediators of intercellular communication. Exosomes circulate in bodily fluids, transferring substances and information between cells and participating in various physiological and pathological processes [14].

Emerging evidence suggests that exosomal RNAs play significant roles in regulating the progression of DFUs. For instance, Liang et al. demonstrated that exosomal circ_HIPK3 may represent a novel therapeutic approach for DFUs through the miR-20b-5p/Nrf2/VEGFA axis [15]. Similarly, Hu et al. reported that exosomes derived from mesenchymal stem cells (MSCs) accelerated diabetic wound healing by promoting angiogenic function through activation of the PI3K/AKT/eNOS pathway [16]. Additional studies have identified that circ_0084443 is upregulated in DFUs and modulates keratinocyte migration and proliferation [17]. Furthermore, exosomes have been shown to significantly promote survival and inhibit the apoptosis of endothelial cells, fibroblasts, keratinocytes, and neurons in the wound microenvironment [18]. These findings collectively suggest that exosome-related genes (ERGs) likely play crucial roles in regulating the development and progression of DFUs. However, comprehensive studies that investigated the mechanisms of action of ERGs in DFU pathogenesis remain limited, highlighting the need for the systematic exploration of exosome-associated biomarkers in DFUs to identify novel therapeutic targets.

This study integrated transcriptomic data and employed advanced bioinformatics methods to comprehensively explore exosome-associated markers related to DEGs in DFUs. Based on these identified markers, we constructed a risk prediction model for DFUs and further investigated the potential regulatory mechanisms and biological pathways involved. Additionally, we examined the correlation between these markers and immune cell infiltration patterns, predicted potential therapeutic drugs, and performed molecular docking analyses to evaluate the potential of these biomarkers as drug targets. Through this multifaceted approach, we aimed to provide new theoretical support and reference for the development of innovative therapeutic strategies for DFU management.

## 2. Materials and Methods

### 2.1. Data Download

The GSE134431 high-throughput dataset and the GSE80178 microarray dataset were downloaded from the GEO database (https://www.ncbi.nlm.nih.gov/geo/, accessed on 15 March 2025) as the training set and validation set, respectively. The GSE134431 dataset, based on the GPL18573 platform, included a total of 21 skin tissue samples, comprising 13 samples from DFU patients and 8 normal control samples. The GSE80178 dataset, based on the GPL16686 platform, included 9 skin tissue samples, comprising 6 samples from DFU patients and 3 normal control samples. A total of 125 exosome-related genes (ERGs) were obtained from a related publication in PubMed [19] (Supplementary Table S1).

### 2.2. Differential Expression Analysis

To identify the differentially expressed genes (DEGs) between different samples in the training set, “DESeq2” (v 1.40.2) was used to perform differential expression analysis between the DFU and control samples in the training set. The DEGs were obtained based on the thresholds of |log_2_FC| > 0.5 and p.adj < 0.05. A volcano plot of DEGs was generated using the R package “ggplot2” (v 3.4.1); meanwhile, a heatmap of DEGs was created using the R package “ComplexHeatmap” (v 2.14.0) to display the expression profiles of the top 10 upregulated and top 10 downregulated genes.

### 2.3. Identification, Enrichment Analysis, and PPI Network of Candidate Genes

To obtain the DEGs related to exosomes, the R package “VennDiagram” (v 1.7.1) was used to intersect the DEGs with ERGs. The intersecting genes were defined as candidate genes for subsequent analyses. To understand the biological pathways in which the candidate genes might be involved, Gene Ontology (GO) and Kyoto Encyclopedia of Genes and Genomes (KEGG) functional enrichment analyses were performed on the candidate genes using the “clusterProfiler” (v 4.2.2) package, with a threshold of p.adj < 0.05. The GO analysis included three categories: biological process (BP), molecular function (MF), and cellular component (CC). To explore the protein–protein interactions (PPIs) between the candidate genes, they were submitted to the Search Tool for the Retrieval of Interacting Genes/Proteins (STRING) database (https://cn.string-db.org/, accessed on 15 March 2025) with an interaction score (confidence level) set at 0.4 to construct a PPI network, which was then visualized using Cytoscape (v 3.8.2) and its Degree algorithm.

### 2.4. Machine Learning and Expression Validation

To further identify candidate biomarkers, an analysis was conducted based on the candidate genes in the training set of the DFU and control samples. First, the Support Vector Machine–Recursive Feature Elimination (SVM-RFE) algorithm was applied using the R package “e1071” (v 1.7-16) to obtain feature genes 1. Following this, the classification wrapper algorithm based on a random forest was performed using the R package “Boruta” (v 8.0.0) to obtain feature genes 2. The intersection of the genes obtained from both algorithms was taken using the R package “VennDiagram” (v 1.7.1) and named as the candidate biomarkers. Finally, in both the training and validation sets, the expression levels of the candidate biomarkers in the DFU and control samples were compared using the Wilcoxon test (*p* < 0.05), and genes that showed consistent differential expression trends in both datasets were selected and named as biomarkers for the subsequent analysis.

### 2.5. Construction and Evaluation of Nomograms

To assess the predictive value of the biomarkers in the DFUs, a nomogram of the biomarkers was constructed using the R package “rms” (6.8-1) in the training set of the DFU and control samples. Subsequently, to evaluate the accuracy of the nomogram’s predictions, calibration curves were plotted using the R package “regplot” (v 1.1).

### 2.6. Localization, Functional Associations, and Correlation Analysis

To determine the locations of the biomarkers on the chromosomes, the “RCircos” (v 1.2.2) package was utilized to analyze the chromosomal positioning of the biomarkers. Additionally, to gain a deeper understanding of the functions of the biomarkers, the mRNALocater database (http://www.rnalocate.org/, accessed on 15 March 2025) was used to predict the subcellular localization of the biomarkers. Subsequently, to identify the functional similarities between the biomarkers in the DFUs, the R package “GOSemSim” (v 2.33.0) was employed to score the functional similarity. Furthermore, based on the training set of the DFU and control samples, the Spearman correlation between the biomarkers was assessed using the “psych” (v 2.1.6) package with the thresholds of |cor| > 0.3 and *p* < 0.05.

### 2.7. Immune Infiltration Analysis

To investigate the infiltration of immune cells in the DFU and control samples, CIBERSORT (v 0.1.0) was used to analyze the relative infiltration abundance of 22 immune cells in all samples of the training set. Samples with *p* > 0.05 were excluded. The Wilcoxon test was then used to compare the differences in the immune cell infiltration between the DFU and control samples (*p* < 0.05) to identify the differential immune cells. Subsequently, based on the DFU and control samples in the training set, the Spearman correlation between differential immune cells and between the biomarkers and differential immune cells was further assessed using the “psych” (v 2.1.6) package with thresholds of |cor| > 0.3 and *p* < 0.05.

### 2.8. Integrated Pathway Enrichment and Gene Interaction Network Analysis

To understand the biological pathways in which the biomarkers are involved in DFUs, a Spearman correlation analysis was conducted between the biomarkers and all other genes in the training set of the DFU and control samples using the “psych” (v 2.1.6) package. The genes were then ranked based on the correlation coefficients from the largest to the smallest. Subsequently, a GSEA-KEGG analysis was performed with the “clusterProfiler” (v 4.2.2) package to explore the potential functions of the biomarkers based on the criteria of |NES| > 1, FDR < 0.25, and *p* < 0.05. Additionally, to further explore other genes and functions related to the biomarkers, the GeneMANIA database (https://genemania.org, accessed on 15 March 2025) was utilized to predict the genes associated with the functions of the biomarkers and the pathways they were involved in.

### 2.9. Regulatory Network Analysis

To better understand the regulatory effects of upstream regulatory factors on the biomarkers, the NetworkAnalyst database (https://www.networkanalyst.ca/, accessed on 15 March 2025) was used to predict transcription factors (TFs) that could interact with the biomarkers, as well as miRNAs that could interact with the biomarkers, and to construct a TF-mRNA-miRNA prediction network.

### 2.10. Computational Drug Discovery and Molecular Interaction Analysis

To explore potential drugs for the treatment of DFUs, the DGIdb database (http://dgidb.org/, accessed on 15 March 2025) was utilized to search for drugs or molecular compounds that may have regulatory relationships with biomarkers. To gain a deeper understanding of the interaction mechanisms between potential drugs and biomarkers, molecular docking was conducted. Initially, the 3D molecular structures of the active ingredients of potential drugs were obtained from the PubChem database (https://pubchem.ncbi.nlm.nih.gov/, accessed on 15 March 2025). For drugs with only 2D structures, the Chem3D tool was used to convert the 2D structures into 3D. Subsequently, the structural data of the proteins corresponding to the biomarkers were retrieved from the UniProt database (https://www.uniprot.org/, accessed on 15 March 2025). Finally, the CD-Dock2 online website (https://cadd.labshare.cn/cb-dock2/php/index.php, accessed on 15 March 2025) was employed to perform in silico molecular docking of the receptor proteins and drug ligands.

### 2.11. RT-qPCR Experiment

To further verify whether the expression of biomarkers in the DFU and control samples was consistent with bioinformatics analysis, we collected five skin tissue samples each from the DFU and control patients at Beijing Shijitan Hospital to detect the expression levels of biomarkers in the samples. All the participants provided written informed consent, and the study was approved by the Ethics Committee of Beijing Shijitan Hospital, Capital Medical University (Approval Number: IIT2025-002-002). RNA was extracted from the samples using TRIzol reagent (Vazyme Biotech, R401-01, Nanjing, Jiangsu Province, China), reverse transcription was performed using the Hifair^®^ III 1st Strand cDNA Synthesis SuperMix for qPCR kit (Yeasen Biotechnology, 11141ES60, Shanghai, China), and primers were synthesized by Shengong Biotech Co., Ltd (Shanghai, China). The expression levels of the biomarkers were calculated using the 2^−ΔΔCt^ method from three independent replicates of RT-qPCR experiments with GAPDH as the reference gene, and the differences in biomarker expression between the DFU and control samples were analyzed using Student’s *t*-test in GraphPad Prism 10 software (*p* < 0.05). The experimental reaction system, conditions, and related primer sequences are referred to in Supplementary Table S2.

### 2.12. Statistical Analysis

All statistical analyses were performed using R statistical software (v 4.2.2). Differences between the two groups were compared using the Wilcoxon rank-sum test (*p* < 0.05). In the RT-qPCR analysis, statistical comparisons were made using the t-test (*p* < 0.05). In the violin plots, box plots, and heatmaps, **** indicates *p* < 0.0001, *** indicates *p* < 0.001, ** indicates *p* < 0.01, * indicates *p* < 0.05, and ns indicates *p* > 0.05.

## 3. Results

### 3.1. Screening of Differential Genes, Identification and Functional Enrichment of Candidate Genes, and PPI Network Analysis

**Figure 1:**
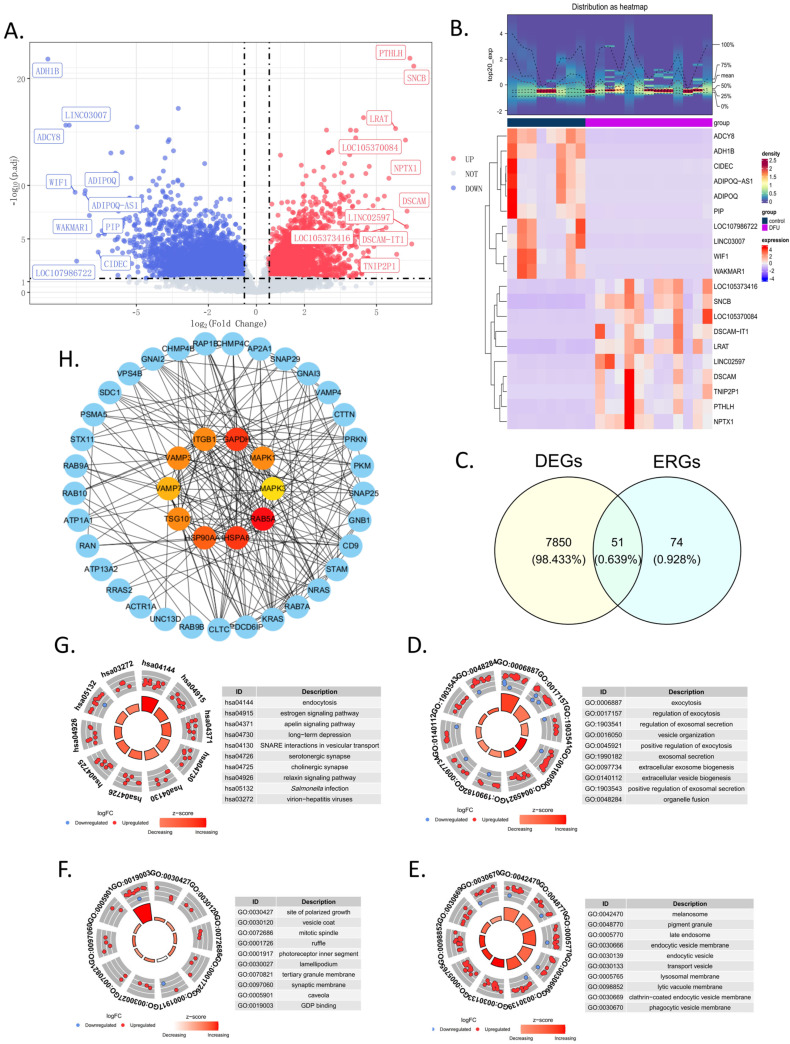
Screening and functional analysis of 51 in candidate genes. (**A**) The volcano map showing the upregulated or downregulated differential genes in the GSE134431 dataset. (**B**) The heatmap of the 7901 DEGs in the GSE134431 dataset. (**C**) Venn diagram showing the 51 common DEGs in GSE134431 and the ERGs, which were named candidate genes. (**D**–**F**) The GO enrichment analysis of the 51 candidate genes: BP, biological process (**D**); CC, cellular components (**E**); MF, molecular function (**F**). (**G**) KEGG enrichment analysis. (**H**) Protein–protein interaction (PPI) network constructed using the STRING database. The inner circle consists of the top 10 genes calculated by degree using Cytoscape; the outer circle shows the 51 candidate genes.

In the training set, a total of 7901 DEGs were identified between the DFU group and the control group, including 3528 upregulated genes and 4373 downregulated genes (Figure 1A). Additionally, to further illustrate the expression levels of these DEGs, a heatmap of their expressions was generated (Figure 1B). By intersecting the 7901 DEGs with the 125 ERGs, 51 candidate genes were obtained (Figure 1C and Supplementary Table S3). These candidate genes were enriched in 542 GO terms (Supplementary Table S4) and 118 KEGG pathways (Supplementary Table S5). Specifically, there were 389 terms in BP, 101 in CC, and 52 in MF. In the GO enrichment analysis, the BP terms were mainly related to exocytosis, the regulation of exocytosis, the regulation of exosomal secretion, vesicle organization, the positive regulation of exocytosis, and exosomal secretion (Figure 1D). For CC, the main enrichments were in the melanosome, pigment granule, late endosome, endocytic vesicle membrane, endocytic vesicle, and transport vesicle (Figure 1E). In terms of MF, the enrichments were primarily in the site of polarized growth, vesicle coat, mitotic spindle, ruffle, photoreceptor inner segment, and lamellipodium (Figure 1F). The top KEGG pathways included endocytosis, the estrogen signaling pathway, the apelin signaling pathway, long-term depression, SNARE interactions in vesicular transport, and serotonergic synapse (Figure 1G). The PPI network indicates that inner-circle genes, such as GAPDH, HSPA8, and RAB5A, play key roles in the network and may have significant roles in biological processes (Figure 1H).

### 3.2. Identification of Biomarkers

**Figure 2:**
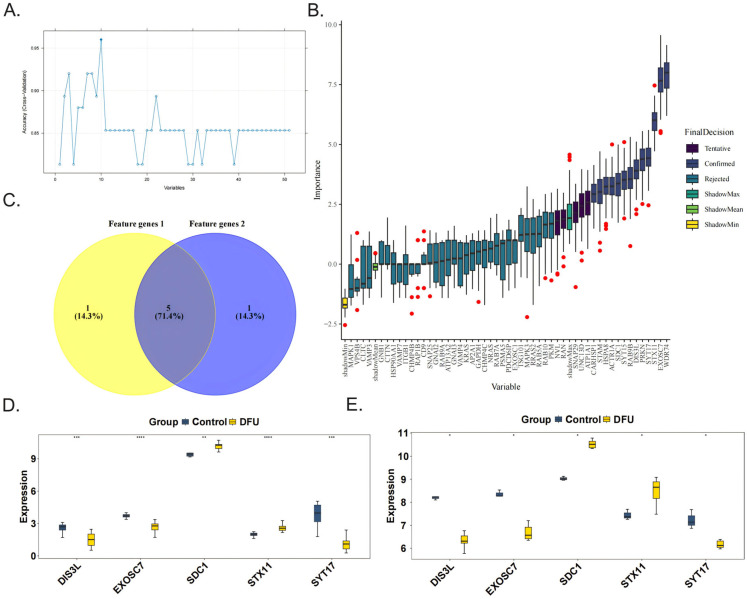
Identification and validation of the 5 biomarkers. The feature genes 1 and feature genes 2 were obtained separately from 51 candidate genes by the SVM-RFE algorithm (**A**) and the Boruta algorithm, the red dots indicate outlier importance values, defined as scores lying beyond 1.5 × IQR from the first or third quartile (**B**). (**C**) The five biomarkers (DIS3L, EXOSC7, SDC1, STX11, SYT17) were identified by the intersection of the two feature genes sets. The validations of the 5 biomarkers in the GSE134431 dataset (**D**) and GSE80178 dataset (**E**). **** indicates *p* < 0.0001, *** indicates *p* < 0.001, ** indicates *p* < 0.01, * indicates *p* < 0.05, and ns indicates *p* > 0.05.

The SVM-RFE algorithm was used to obtain six feature genes 1, namely, DIS3L, EXOSC7, SDC1, STX11, SYT17, and UNC13D (Figure 2A). The Boruta algorithm was then applied to acquire six feature genes 2, which were CARHSP1, DIS3L, EXOSC7, SDC1, STX11, and SYT17 (Figure 2B). After taking the intersection, five candidate biomarkers were identified, namely, DIS3L, EXOSC7, SDC1, STX11, and SYT17 (Figure 2C). When comparing the expression trends of the candidate biomarkers, it was found that all five genes showed consistent trends and significant differences (*p* < 0.05) in both the training and validation sets, leading to their definition as biomarkers. Among them, the expression levels of DIS3L, EXOSC7, and SYT17 decreased, while the expression levels of SDC1 and STX11 increased (Figure 2D,E).

### 3.3. Construction of Diagnostic Models

**Figure 3:**
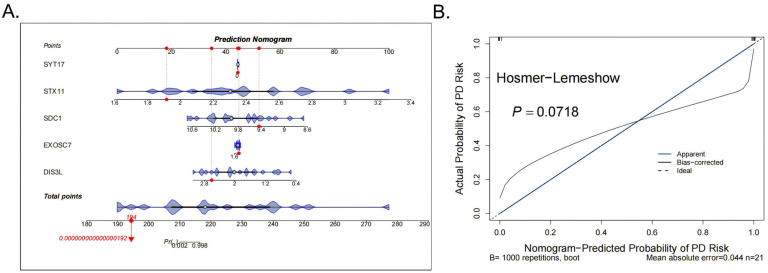
Construction and evaluation of the nomogram. (**A**) The nomogram for the 5 biomarkers of the diagnostic models, the red dots indicate the position of a specific individual or sample on each variable in terms of its corresponding score. (**B**) The calibration curve results of the model.

Subsequently, to improve the accuracy of the DFU diagnosis, a nomogram was constructed for the five biomarkers (SYT17, STX11, SDC1, EXOSC7, DIS3L) (Figure 3A). The calibration curve showed no significant difference between the predicted probabilities and observed outcomes (Hosmer–Lemeshow test *p* = 0.0718), indicating acceptable model calibration. Furthermore, the model achieved a low mean absolute error (MAE = 0.044), supporting its practical reliability (Figure 3B).

### 3.4. Chromosome and Subcellular Localization and Friends and Correlation Analyses

**Figure 4:**
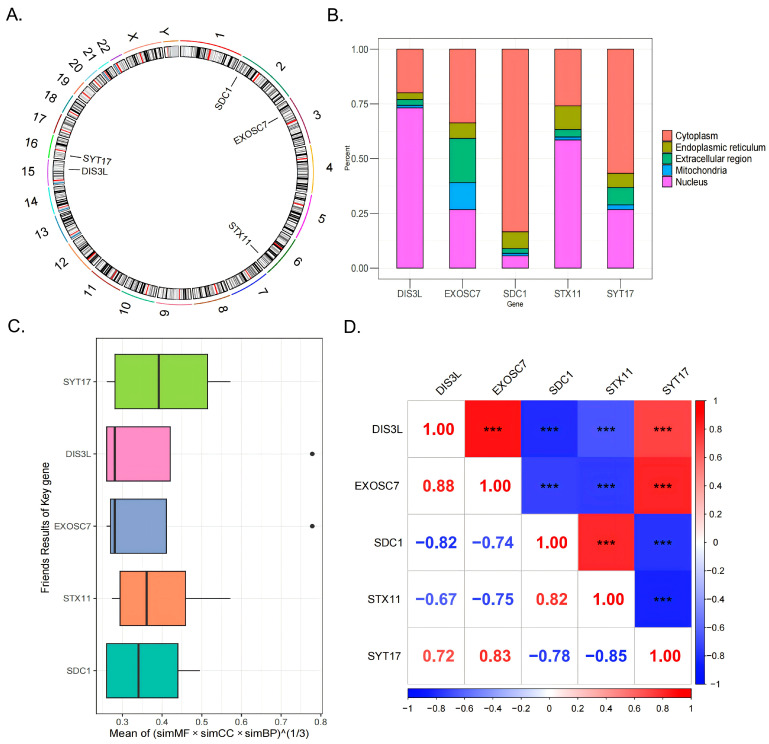
The 5 biomarkers’ chromosomal localization (**A**) and subcellular localization (**B**). (**C**) The Friends analysis boxplot, the black dots indicate the genes of the outliers exhibit significant differences from other genes in terms of the comprehensive scores of functional similarity across these three categories. (**D**) The genes correlation analysis results. *** indicates *p* < 0.001.

Chromosomal localization analysis revealed that SDC1 was located on chromosome 2, EXOSC7 on chromosome 3, STX11 on chromosome 6, DIS3L on chromosome 15, and SYT17 on chromosome 16 (Figure 4A). Subcellular localization analysis indicated that DIS3L and STX11 were primarily concentrated in the nucleus, followed by the cytoplasm; EXOSC7 had a relatively even distribution, with a focus on the cytoplasm and nucleus; and SDC1 and SYT17 were mainly concentrated in the cytoplasm, followed by the nucleus (Figure 4B). These distribution patterns suggest that DIS3L, EXOSC7, SDC1, STX11, and SYT17 might play significant roles in the nucleus and cytoplasm, while their distributions in the mitochondria, endoplasmic reticulum, and extracellular regions were relatively limited, indicating potentially lesser functionality in these areas. The Friends analysis boxplot showed that the functional similarity score distribution for SYT17 was broad, with a high median, suggesting a higher likelihood of functional similarity with other genes (Figure 4C). In the gene correlation analysis, strong positive correlations (|cor| > 0.7, *p* < 0.0001) were observed between DIS3L and EXOSC7, DIS3L and SYT17, EXOSC7 and SYT17, and SDC1 and STX11. In contrast, strong negative correlations (|cor| > 0.7, *p* < 0.0001) were found between DIS3L and SDC1, DIS3L and STX11, EXOSC7 and SDC1, EXOSC7 and STX11, SDC1 and SYT17, and STX11 and SYT17 (Figure 4D).

### 3.5. Immunological Feature Analysis

**Figure 5:**
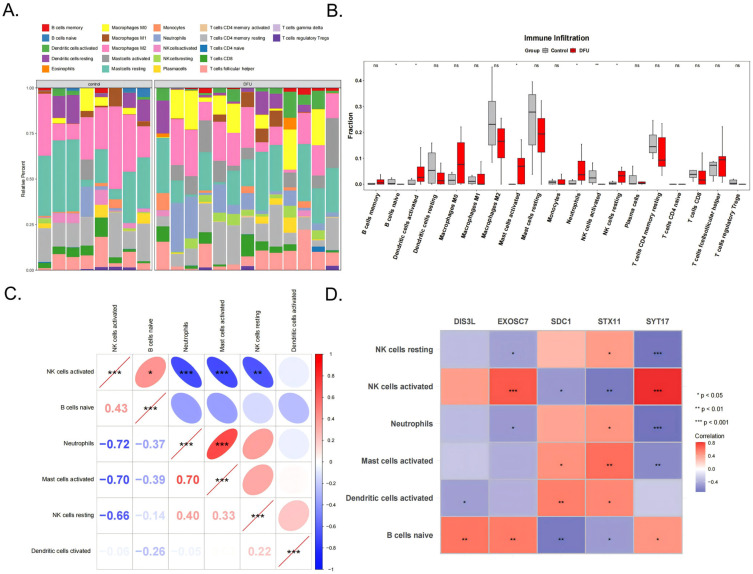
The 22 types of immune cells (**A**) and the 6 significant different types shown between the DFU and control groups (**B**). (**C**) Correlation analysis between the 6 types of immune cells. (**D**) The correlation analysis of the 5 biomarkers and 6 immune cells. *** indicates *p* < 0.001, ** indicates *p* < 0.01, * indicates *p* < 0.05, and ns indicates *p* > 0.05.

The immune cell infiltration patterns in the DFU patients affected wound healing. The analysis results of the infiltration abundance of 22 immune cell types in the DFU patients are shown in Figure 5A. Six cell types showed significant differences between the DFU and control groups (*p* < 0.05), including activated NK cells, naive B cells, activated dendritic cells, activated mast cells, neutrophils, and resting NK cells (Figure 5B). Among them, neutrophils were significantly positively correlated with activated mast cells (cor = 0.70, *p* < 0.001) and activated NK cells were significantly positively correlated with naive B cells (cor = 0.43, *p* < 0.05). Activated NK cells showed significant negative correlations with neutrophils (cor = −0.72, *p* < 0.001), activated mast cells (cor = −0.70, *p* < 0.001), and resting NK cells (cor = −0.66, *p* < 0.001) (Figure 5C). Additionally, activated NK cells showed extremely significant positive correlations with EXOSC7 (cor = 0.70, *p* < 0.001) and SYT17 (cor = 0.81, *p* < 0.001), while SYT17 showed extremely significant negative correlations with resting NK cells (cor = −0.68, *p* < 0.001) and neutrophils (cor = −0.70, *p* < 0.001) (Figure 5D).

### 3.6. Gene Set Enrichment Analysis and GeneMANIA Analysis

**Figure 6:**
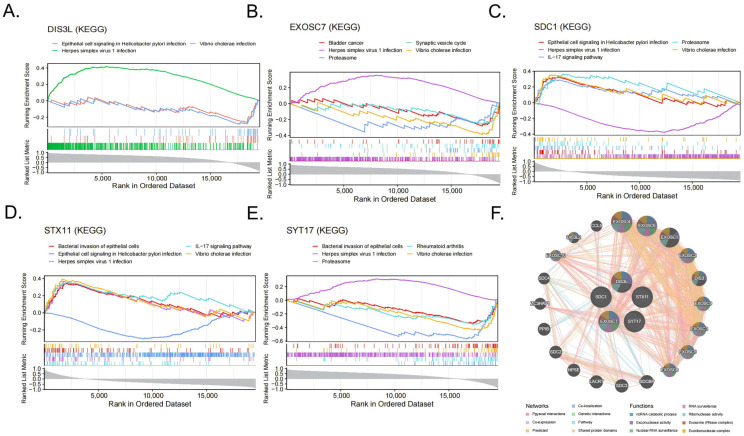
(**A**–**E**) The items enriched by GSEA-KEGG of the 5 biomarkers. (**F**) Networks and functions of the 5 biomarkers with their related genes were analyzed via GeneMANIA.

The GSEA-KEGG enrichment analysis results indicate that DIS3L was mainly enriched in herpes simplex virus 1 infections, epithelial cell signaling in *Helicobacter pylori* infections, and *Vibrio cholerae* infections (Figure 6A); EXOSC7 was mainly enriched in herpes simplex virus 1 infections, *Vibrio cholerae* infections, the proteasome, the synaptic vesicle cycle, bladder cancer, and the VEGF signaling pathway (Figure 6B); SDC1 was mainly enriched in herpes simplex virus 1 infections, epithelial cell signaling in *Helicobacter pylori* infections, the IL-17 signaling pathway, the proteasome, *Vibrio cholerae* infections, and the bacterial invasion of epithelial cells (Figure 6C); STX11 was mainly enriched in the IL-17 signaling pathway, epithelial cell signaling in *Helicobacter pylori* infections, herpes simplex virus 1 infections, the bacterial invasion of epithelial cells, *Vibrio cholerae* infections, and the HIF-1 signaling pathway (Figure 6D); SYT17 was mainly enriched in the proteasome, rheumatoid arthritis, herpes simplex virus 1 infections, *Vibrio cholerae* infections, the bacterial invasion of epithelial cells, and the IL-17 signaling pathway (Figure 6E). In summary, all five biomarkers were enriched in the *Vibrio cholerae* infection and herpes simplex virus 1 infection pathways. The GeneMANIA network displays the interaction network of the five biomarkers and their 20 related genes (Figure 6F). These functions were primarily involved in RNA metabolism, surveillance, and degradation processes, such as ncRNA catabolic process, exonuclease activity, and nuclear RNA surveillance.

### 3.7. TF-miRNA-mRNA Regulatory Network

**Figure 7:**
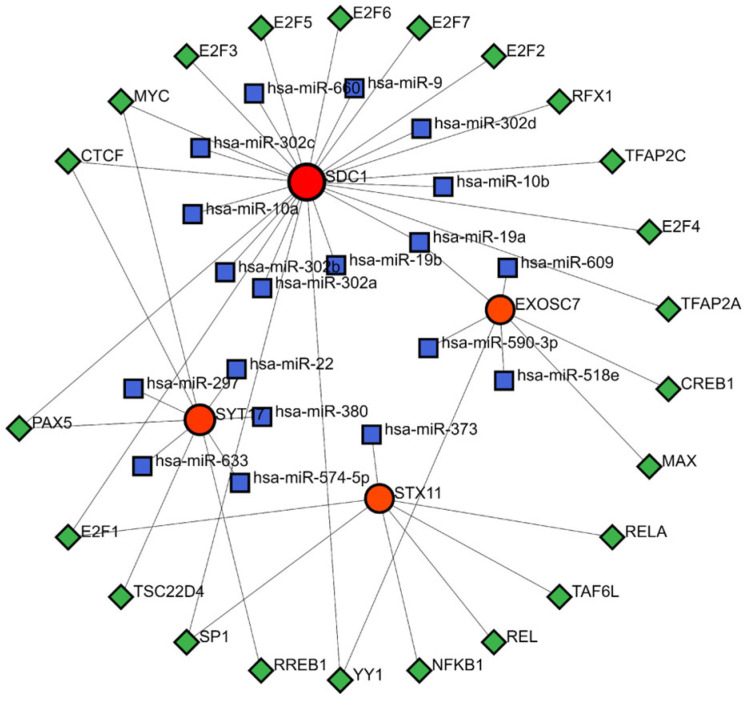
TF-miRNA-mRNA regulatory network. Red represents mRNA, blue represents miRNA, and green represents TF.

The TF-mRNA-miRNA prediction network comprised 4 biomarkers, 23 TFs, and 19 miRNAs (Figure 7). Within this network, hsa-miR-19a was able to interact with SDC1 and EXOSC7 to regulate the gene expression. Additionally, the TFs MYC, CTCF, and PAX5 could bind to the DNA of SDC1 and SYT17 genes, while E2F1 could bind to the DNA of the SDC1 and STX17 genes. Similarly, SP1 and YY1 could bind to the DNA of the SDC1 and EXOSC7 genes, modulating the transcription processes of these genes.

### 3.8. Drug Prediction and Molecular Docking

**Figure 8:**
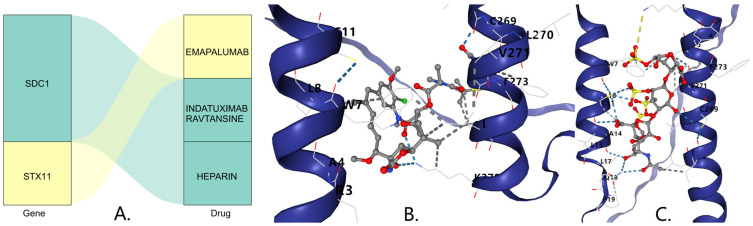
(**A**) Prediction of key gene-targeted (SDC1) drugs. The three-dimensional molecular structure of the Indatuximab ravtansine (**B**) and Heparin (**C**).

In the DSigDB database, SDC1 was predicted to be associated with the drugs Indatuximab ravtansine and Heparin, while STX11 was predicted to be associated with the drug Emapalumab (Figure 8A). The molecular docking results indicate that the binding free energy of Indatuximab ravtansine with the SDC1 target was −6.7 kcal/mol (Figure 8B, Table 1), which primarily bound with sites A4, C269, L270, etc. The binding free energy of Heparin with the SDC1 target was −5.4 kcal/mol (Figure 8C, Table 1), which mainly bound with sites A14, C269, L17, etc. This indicates that both Indatuximab ravtansine and Heparin had strong binding affinities with SDC1.

### 3.9. Clinical Sample Experimental Validation

**Figure 9:**
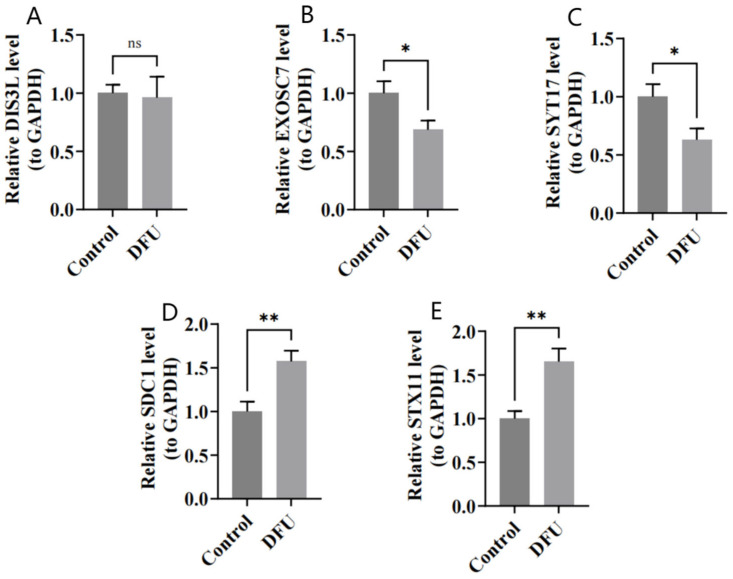
(**A**–**E**) Relative expressions of DIS3L, EXOSC7, SDC1, STX11, and SYT17 by RT-qPCR analysis. ** indicates *p* < 0.01, * indicates *p* < 0.05, and ns indicates *p* > 0.05.

The RT-qPCR results show that DIS3L, EXOSC7, and SYT17 were underexpressed in the DFU group (Figure 9A–C), while SDC1 and STX11 were overexpressed in the DFU group (Figure 9D,E), which was consistent with the bioinformatics analysis. The expression differences were statistically significant (*p* < 0.05), except for DIS3L.

## 4. Discussion

Diabetic foot ulcers represent one of the most common and economically burdensome complications of diabetes mellitus [20]. Despite recent efforts to investigate DFU pathogenesis using bioinformatics approaches, studies often lack specificity and corresponding clinical validation, resulting in limited understanding of the underlying mechanisms. Therefore, deeper exploration of DFU pathogenesis is essential to identify potential therapeutic targets and provide additional treatment options. Lower extremity arterial disease, a major pathological contributor to DFUs, is frequently accompanied by vascular stenosis or occlusion. While Western treatments, such as antiplatelet and revascularization therapies, are foundational, they are often limited by indications and high recurrence rates. Traditional Chinese Medicine, guided by principles of “activating blood and unblocking collaterals,” offers systemic modulation and microcirculatory improvement. Herbs like Danshen and Chuanxiong, which are key ingredients in Guanxinning Tablets, have demonstrated antioxidative, anti-inflammatory, and vasodilatory effects, suggesting therapeutic potential in lower-extremity-arterial-disease-related DFUs under the “treating different diseases with the same method” theory. This integrative approach warrants further exploration in future DFU management strategies.

This study pioneered an innovative approach by integrating exosome-associated genes with differentially expressed genes in diabetic foot ulcers. We identified 51 candidate genes through this intersection and subsequently employed two distinct machine learning methods to screen for key genes. Five core biomarkers—DIS3L, EXOSC7, SDC1, STX11, and SYT17—were identified and validated in both the training and validation datasets. These biomarkers were then subjected to comprehensive functional enrichment, drug prediction, and molecular docking analyses, and ultimately validated in clinical samples through RT-qPCR.

The functional enrichment analyses yielded significant insights into the biological mechanisms underlying DFU pathogenesis. The GO analysis revealed that the 51 candidate genes were predominantly enriched in biological processes related to exocytosis, vesicle organization, and exosomal secretion. These processes play crucial roles in cellular communication, particularly in wound healing, where coordinated interactions between different cell types are essential. The cellular component analysis highlighted enrichment in melanosomes, pigment granules, late endosomes, and transport vesicles, suggesting the involvement of multiple cellular compartments in exosome biogenesis and release during DFU development. KEGG pathway analysis further demonstrated enrichment in endocytosis, estrogen signaling, apelin signaling, and SNARE interactions in vesicular transport, indicating that vesicular trafficking and hormonal regulation may be critical in DFU pathophysiology. The PPI network analysis identified central nodes, such as GAPDH, HSPA8, and RAB5A, which likely serve as hub genes coordinating the complex interplay between exosome-related processes in DFUs.

Numerous studies have demonstrated the therapeutic efficacy of exosomes in DFUs [21]. Building on this foundation, we focused on five biomarkers—DIS3L, EXOSC7, SDC1, STX11, and SYT17—to gain deeper insights into DFU pathogenesis. While previous studies have proposed exosomal circRNAs as novel biomarkers for early DFU diagnosis [22], they primarily extracted biomarkers from serum. We contend that biomarkers derived directly from ulcer tissues may provide more accurate diagnostic indicators, which informed our approach to construct a diagnostic model using tissue-derived biomarkers and validate them with clinical tissue samples. Furthermore, a canonical marker of angiogenesis called VEGFA was consistently downregulated in the DFUs due to impaired vascular repair. While VEGFA excels in predicting poor wound perfusion, our nomogram (which combined all five biomarkers) achieved superior calibration by integrating multiple pathways [23]. Notably, SDC1′s association with the IL-17 pathway may complement VEGFA’s angiogenic focus by addressing inflammatory dysregulation. HIF1A was upregulated in early DFUs but lost predictive power in chronic ulcers due to persistent hypoxia. In contrast, STX11 and SYT17 (linked to microbial response and vesicular transport) may better distinguish acute vs. chronic phases, as they correlate dynamically with immune cell infiltration (e.g., NK cells, neutrophils) [24].

GSEA functional enrichment analysis revealed that DIS3L, a key exosome-associated gene, plays an important role in microbial infection and host cell responses in DFU patients. Previous studies have established DIS3L’s significant role in RNA degradation and metabolic regulation within cells [25], suggesting it may participate in DFU pathology by modulating host cell metabolism and immune responses. However, our clinical sample validation did not demonstrate the significant differential expression of DIS3L, possibly due to its limited expression in ulcer tissues. EXOSC7, another exosome-associated gene, participates in RNA degradation, influencing cell cycle regulation and protein synthesis [26]. EXOSC7 has been linked to exosomopathies, which are diseases that typically manifest as neurological or developmental abnormalities [27]. Our study found that EXOSC7 was associated with proteasome pathways that underwent activity changes under hyperglycemic conditions, potentially leading to microvascular damage [28]—a key factor contributing to poor healing in diabetic foot ulcers. SDC1 (Syndecan-1), a membrane-bound heparan sulfate proteoglycan, binds to extracellular matrix proteins and regulates cell adhesion and migration—which are processes critical for wound healing [29]. Previous research has shown that SDC1 can bind to various signaling molecules, including growth factor receptors that may activate the PI3K/AKT/mTOR signaling pathway. Our study indicates that SDC1 was significantly associated with the IL-17 signaling pathway, which can activate the PI3K/AKT/mTOR pathway [30] and promote Th17 cell differentiation and function [31]. While Th17 cells play crucial roles in defending against pathogens, their overactivation may lead to tissue damage. Diabetic patients often exhibit chronic inflammatory states, and Th17 cells may influence DFU development and healing by promoting this inflammatory condition [32]. SYT17 (Synaptotagmin-17) is an important vesicle-associated protein that is widely involved in vesicular transport and intercellular signaling [33]. Our findings suggest that SYT17 may be significantly related to regulating epithelial cell barrier function, involving Rho family GTPases and MAPK pathways, which play important roles in DFU pathogenesis, particularly in cell migration, tissue repair, and inflammation regulation [34]. While direct evidence linking SYT17 to these pathways is currently lacking, SYT17′s role in regulating neuronal membrane trafficking and signal transduction may indirectly influence these pathways. STX11 (Syntaxin-11) is an important membrane protein that influences microbial pathogens and immune responses by regulating TLR4 transport [35]. This may be related to our findings of enrichment in regulating epithelial cell barrier function and the IL-17 pathway [36], suggesting its role in immune response and inflammation regulation may indirectly affect the development and healing of diabetic foot ulcers. Notably, all five biomarkers showed enrichment in the herpes simplex virus type 1 (HSV-1) infection pathways. Research has established connections between HSV-1 infection and diabetes, with infection activating cellular PI3K/Akt and p38 MAPK signaling pathways [37]. We speculate this may be related to the SYT17-associated MAPK pathway and the SDC1-involved IL-17 pathway, though further research is needed to validate these connections.

Our CIBERSORT analysis results revealed significant differences in immune cell infiltration patterns between DFU patients and controls, with significant correlations between immune cell levels and biomarker expression. Figure 5D indicates that SYT17 was significantly negatively correlated with resting NK cells, and with a decreased SYT17 expression in DFU patients, the resting NK cells tended to increase. Conversely, SYT17 showed a significant positive correlation with activated NK cells, suggesting a decrease in the activated NK cells. Similar results were observed with STX11, potentially resulting in impaired immune function and increased infection risk [38]. This intriguing finding suggests that SYT17 and STX11 may inhibit the transition of resting NK cells to an activated state, though this complex process requires further investigation. Additionally, we found that EXOSC7 was significantly positively correlated with naive B cells. In DFU patients, decreased EXOSC7 expression is associated with reduced naive B cell numbers, with similar results for SDC1. This leads to fewer mature B cells during wound healing, and research has shown [39] that increased mature B cells can accelerate diabetic wound healing. Figure 5C indicates that the activated NK cells were significantly positively correlated with the naive B cells in the DFU patients, where both immune cell types showed decreased expressions. Therefore, we hypothesize that SYT17, STX11, EXOSC7, and SDC1 participate in the immune microenvironment by influencing the expression of these two cell types through intercellular signaling pathways, offering new perspectives for DFU treatment.

Our construction of a TF-miRNA-mRNA regulatory network revealed multilayered regulatory mechanisms of biomarkers in the DFUs. Figure 7 shows that SDC1 had the most associations with TFs and miRNAs between the biomarkers. Research has confirmed that E2F1 enhances M2 phenotype macrophage polarization, accelerating wound healing [40], while other studies suggest SDC1 may regulate the transition between M1 and M2 macrophages [41]. This suggests that E2F1 might influence wound healing by targeting SDC1 to affect the M2 macrophage expression. We also observed that hsa-miR-19a/b targeted SDC1 to regulate its expression. Studies have shown that miR-19b can target and downregulate SDC1 in lung endothelial cells [42], and miR-19a/b promotes wound healing by inhibiting TLR3-mediated NF-κB activation through the regulation of SHCBP1 and SEMA7A genes, reducing the inflammatory chemokines and cytokines produced by keratinocytes [43]. This coordinated regulation between miRNAs and transcription factors provides new perspectives on DFU pathology and potential targets for miRNA and transcription-factor-based therapeutic strategies.

Through drug prediction and molecular docking analyses, we explored potential therapeutic drugs targeting DFU-related biomarkers. Previous research has confirmed that Indatuximab ravtansine inhibits cancer cell growth by targeting SDC1 [44]. Our molecular docking results show that Indatuximab ravtansine demonstrated strong binding affinity with SDC1 (−6.7 kcal/mol), where it primarily bound at sites A4, C269, L270, etc. Similarly, Heparin also exhibited a notable binding affinity with SDC1 (−5.4 kcal/mol), mainly at sites A14, C269, L17, etc. These findings suggest that these drugs could potentially modulate the SDC1 activity in DFUs, offering promising therapeutic avenues. While Indatuximab ravtansine is primarily studied in cancer contexts, our findings highlight its potential application in DFU treatment by targeting SDC1-mediated signaling pathways, particularly those involved in inflammation and wound-healing processes. Our RT-qPCR validation in clinical samples confirmed the differential expression of all five biomarkers. DIS3L, EXOSC7, and SYT17 were underexpressed in the DFU samples, while SDC1 and STX11 were overexpressed, which was consistent with our bioinformatics analyses. These experimental validations provide robust support for our computational findings and strengthen the potential clinical relevance of these biomarkers. Moreover, the consistent expression patterns across the computational and experimental approaches suggest that these biomarkers may serve as reliable diagnostic indicators and therapeutic targets for DFUs.

The nomogram that combined these biomarkers demonstrated robust diagnostic accuracy (MAE = 0.044). These markers could be incorporated into liquid biopsy panels (e.g., serum or wound exosome profiling) to complement existing diagnostic tools. Given their association with immune modulation, these biomarkers could be adapted into rapid multiplex assays (e.g., ELISA) to stratify patients based on infection risk or healing potential. Then, the TF-miRNA-mRNA network revealed regulatory nodes (e.g., miR-19a/b targeting SDC1). miRNA-based therapies or small-molecule inhibitors (e.g., targeting IL-17 or PI3K/AKT pathways) could be tailored to individual biomarker profiles. The strong correlations between biomarkers (e.g., SYT17 and NK cells) suggest utility in tracking immune dysfunction. The regular profiling of these markers could guide immunotherapy or antimicrobial strategies. The longitudinal assessment of EXOSC7 (linked to proteasome dysfunction) and STX11 (associated with microbial response) might predict a response to debridement or advanced dressings.

This study had several limitations that should be acknowledged. First, although the candidate biomarkers were screened by intersecting DEGs with previously published exosome-related gene sets, our study did not include experimental validation to demonstrate the actual packaging of these biomarkers into exosomes (e.g., via ultracentrifugation-based EV isolation, nanoparticle tracking analysis, or Western blotting for canonical EV markers). Therefore, we cannot definitively conclude that these genes are functionally active within exosomal vesicles. Further studies involving exosome isolation and proteomic validation are warranted to confirm their presence and roles within EVs in the DFU microenvironment Second, while we identified potential mechanisms and drug targets, experimental validation of the proposed pathways and drug efficacies in appropriate DFU models is necessary. Third, the complex interactions between exosomes, immune cells, and wound-healing processes warrant further investigation through functional studies. Future research should focus on elucidating the precise mechanisms by which these biomarkers influence DFU pathogenesis and healing, as well as developing targeted therapies based on these findings.

In conclusion, this study identified and validated five exosome-associated biomarkers with significant potential for DFU diagnosis and treatment. Our comprehensive bioinformatics analyses and experimental validation provide novel insights into the complex molecular mechanisms underlying DFU pathogenesis, particularly highlighting the roles of exosome-mediated signaling and immune cell interactions. The identification of potential therapeutic drugs through computational approaches offers promising avenues for developing targeted DFU treatments. These findings collectively contribute to our understanding of DFU pathophysiology and may guide future clinical applications aimed at improving outcomes for patients with this debilitating complication of diabetes.

## Figures and Tables

**Table 1:** Minimum binding energy of biomarkers docking with drugs.

Gene	Medicine	Vina Score (kcal/mol)
SDC1	Indatuximab ravtansine	−6.7
SDC1	Heparin	−5.4

## Data Availability

The data supporting the findings of this study are available from the corresponding author upon reasonable request. The data are not publicly available due to privacy and ethical restrictions.

## Supplementary Materials

The following supporting information can be downloaded from https://www.mdpi.com/article/10.3390/biomedicines13071687/s1, Table S1: The 125 exosome-related genes from the literature and databases. Table S2: The RT-qPCR experimental reaction system, conditions, and related primer sequences. Table S3: The 51 candidate genes. Table S4: The 542 GO terms of the 51 candidate genes. Table S5: The 118 KEGG pathways of the 51 candidate genes.

## Abbreviations

DFU	Diabetic foot ulcer
DEGs	Differentially expressed genes
ERGs	Exosome-related genes
LD	Linear dichroism
GO	Gene Ontology
KEGG	Kyoto Encyclopedia of Genes and Genomes
PPI	Protein–protein interactions
BP	Biological process
MF	Molecular function
CC	Cellular component
SVM-RFE	Support Vector Machine–Recursive Feature Elimination
TFs	Transcription factors
GESA	Gene set enrichment analysis

## References

[1] Lin X., Xu Y., Pan X., Xu J., Ding Y., Sun X., Song X., Ren Y., Shan P.F. (2020). Global, regional, and national burden and trend of diabetes in 195 countries and territories: An analysis from 1990 to 2025. Sci. Rep..

[2] Bolton L. (2022). Diabetic foot ulcer: Treatment challenges. Wounds.

[3] Rodrigues B.T., Vangaveti V.N., Urkude R., Biros E., Malabu U.H. (2022). Prevalence and risk factors of lower limb amputations in patients with diabetic foot ulcers: A systematic review and meta-analysis. Diabetes Metab. Syndr..

[4] Fu X.L., Ding H., Miao W.W., Mao C.X., Zhan M.Q., Chen H.L. (2019). Global recurrence rates in diabetic foot ulcers: A systematic review and meta-analysis. Diabetes Metab. Res. Rev..

[5] Yarahmadi A., Modaghegh M.-H.S., Mostafavi-Pour Z., Azarpira N., Mousavian A., Bonakdaran S., Jarahi L., Samadi A., Peimani M., Alamdari D.H. (2021). The effect of platelet-rich plasma-fibrin glue dressing in combination with oral vitamin E and C for treatment of non-healing diabetic foot ulcers: A randomized, double-blind, parallel-group, clinical trial. Expert. Opin. Biol. Ther..

[6] Wang X., Yuan C.X., Xu B., Yu Z. (2022). Diabetic foot ulcers: Classification, risk factors and management. World J. Diabetes.

[7] Champlain A.H., DiGiorgio C.M., Zurakowski D., Sakamoto F.H., Anderson R.R. (2022). Wound Healing After Fractional Skin Harvesting. Dermatol. Surg..

[8] Chen X.Y., Jiang W.W., Liu Y.L., Ma Z.X., Dai J.Q. (2022). Anti-inflammatory action of geniposide promotes wound healing in diabetic rats. Pharm. Biol..

[9] Chen L., Zheng B., Xu Y., Sun C., Wu W., Xie X., Zhu Y., Cai W., Lin S., Luo Y. (2023). Nano hydrogel-based oxygen-releasing stem cell transplantation system for treating diabetic foot. J. Nanobiotechnol..

[10] Rego S.M., Snyder M.P. (2019). High Throughput Sequencing and Assessing Disease Risk. Cold Spring Harb. Perspect. Med..

[11] Guo L., Xiao D., Xing H., Yang G., Yang X. (2024). Engineered exosomes as a prospective therapy for diabetic foot ulcers. Burns Trauma.

[12] He X., Kuang G., Wu Y., Ou C. (2021). Emerging roles of exosomal miRNAs in diabetes mellitus. Clin. Transl. Med..

[13] Jella K.K., Nasti T.H., Li Z., Malla S.R., Buchwald Z.S., Khan M.K. (2018). Exosomes, Their Biogenesis and Role in Inter-Cellular Communication, Tumor Microenvironment and Cancer Immunotherapy. Vaccines.

[14] Chang W., Wang J. (2019). Exosomes and Their Noncoding RNA Cargo Are Emerging as New Modulators for Diabetes Mellitus. Cells.

[15] Ma S., Tian Y., Peng J., Chen C., Peng X., Zhao F., Li Z., Li M., Zhao F., Sheng X. (2023). Identification of a small-molecule Tim-3 inhibitor to potentiate T cell-mediated antitumor immunotherapy in preclinical mouse models. Sci. Transl. Med..

[16] Hu Y., Tao R., Chen L., Xiong Y., Xue H., Hu L., Yan C., Xie X., Lin Z., Panayi A.C. (2021). Exosomes derived from pioglitazone-pretreated MSCs accelerate diabetic wound healing through enhancing angiogenesis. J. Nanobiotechnol..

[17] Wang A., Toma M.A., Ma J., Li D., Vij M., Chu T., Wang J., Li X., Landén N.X. (2020). Circular RNA hsa_circ_0084443 Is Upregulated in Diabetic Foot Ulcer and Modulates Keratinocyte Migration and Proliferation. Adv. Wound Care.

[18] Shiekh P.A., Singh A., Kumar A. (2020). Exosome laden oxygen releasing antioxidant and antibacterial cryogel wound dressing OxOBand alleviate diabetic and infectious wound healing. Biomaterials.

[19] Peng W., Bai S., Zheng M., Chen W., Li Y., Yang Y., Zhao Y., Xiong S., Wang R., Cheng B. (2023). An exosome-related lncRNA signature correlates with prognosis, immune microenvironment, and therapeutic responses in hepatocellular carcinoma. Transl. Oncol..

[20] Sa B.C., Maskan Bermudez N., Shimon S.V., Kirsner R.S. (2024). Diabetic Foot Ulcers: A Review of Debridement Techniques. Surg. Technol. Int..

[21] Yang Z., Yang M., Rui S., Hao W., Wu X., Guo L., Armstrong D.G., Yang C., Deng W. (2024). Exosome-based cell therapy for diabetic foot ulcers: Present and prospect. Heliyon.

[22] Li Z., Ren Y., Lv Z., Li M., Li Y., Fan X., Xiong Y., Qian L. (2023). Decrypting the circular RNAs does a favor for us: Understanding, diagnosing and treating diabetes mellitus and its complications. Biomed. Pharmacother..

[23] Xu J., Gao J., Li H., Zhu Z., Liu J., Gao C. (2024). The risk factors in diabetic foot ulcers and predictive value of prognosis of wound tissue vascular endothelium growth factor. Sci. Rep..

[24] Catrina S.B., Zheng X. (2021). Hypoxia and hypoxia-inducible factors in diabetes and its complications. Diabetologia.

[25] Luan S., Luo J., Liu H., Li Z. (2019). Regulation of RNA decay and cellular function by 3′-5′ exoribonuclease DIS3L2. RNA Biol..

[26] Yuan Y., Mao X., Abubakar Y.S., Zheng W., Wang Z., Zhou J., Zheng H., Albuquerque P. (2023). Genome-Wide Characterization of the RNA Exosome Complex in Relation to Growth, Development, and Pathogenicity of Fusarium graminearum. Microbiol. Spectr..

[27] Bressman Z.J., Corbett A.H., Ghalei H. (2025). Built differently or defective: Can RNA exosomopathies cause ribosome heterogeneity?. Philos. Trans. R. Soc. Lond. B Biol. Sci..

[28] Svikle Z., Peterfelde B., Sjakste N., Baumane K., Verkauskiene R., Jeng C.-J., Sokolovska J. (2022). Ubiquitin-proteasome system in diabetic retinopathy. PeerJ.

[29] Frontiers Production O. (2022). Erratum: The role and therapeutic value of syndecan-1 in cancer metastasis and drug resistance. Front. Cell Dev. Biol..

[30] Varshney P., Saini N. (2018). PI3K/AKT/mTOR activation and autophagy inhibition plays a key role in increased cholesterol during IL-17A mediated inflammatory response in psoriasis. Biochim. Et Biophys. Acta Mol. Basis Dis..

[31] Lin Y.W., Li X.X., Fu F.H., Liu B., Xing X., Qi R., Ma L. (2022). Notch1/Hes1-PTEN/AKT/IL-17A feedback loop regulates Th17 cell differentiation in mouse psoriasis-like skin inflammation. Mol. Med. Rep..

[32] Holl J., Kowalewski C., Zimek Z., Fiedor P., Kaminski A., Oldak T., Moniuszko M., Eljaszewicz A. (2021). Chronic Diabetic Wounds and Their Treatment with Skin Substitutes. Cells.

[33] MacDougall D.D., Lin Z., Chon N.L., Jackman S.L., Lin H., Knight J.D., Anantharam A. (2018). The high-affinity calcium sensor synaptotagmin-7 serves multiple roles in regulated exocytosis. J. Gen. Physiol..

[34] Guo Y.J., Pan W.W., Liu S.B., Shen Z.F., Xu Y., Hu L.L. (2020). ERK/MAPK signalling pathway and tumorigenesis. Exp. Ther. Med..

[35] Ruhl D.A., Bomba-Warczak E., Watson E.T., Bradberry M.M., Peterson T.A., Basu T., Frelka A., Evans C.S., Briguglio J.S., Basta T. (2019). Synaptotagmin 17 controls neurite outgrowth and synaptic physiology via distinct cellular pathways. Nat. Commun..

[36] Kang Y., Su G., Sun J., Zhang Y. (2018). Activation of the TLR4/MyD88 signaling pathway contributes to the development of human hepatocellular carcinoma via upregulation of IL-23 and IL-17A. Oncol. Lett..

[37] Tang Q., Luan F., Yuan A., Sun J., Rao Z., Wang B., Liu Y., Zeng N. (2022). Sophoridine Suppresses Herpes Simplex Virus Type 1 Infection by Blocking the Activation of Cellular PI3K/Akt and p38 MAPK Pathways. Front. Microbiol..

[38] Dean I., Lee C.Y.C., Tuong Z.K., Li Z., Tibbitt C.A., Willis C., Gaspal F., Kennedy B.C., Matei-Rascu V., Fiancette R. (2024). Rapid functional impairment of natural killer cells following tumor entry limits anti-tumor immunity. Nat. Commun..

[39] Sîrbulescu R.F., Boehm C.K., Soon E., Wilks M.Q., Ilieş I., Yuan H., Maxner B., Chronos N., Kaittanis C., Normandin M.D. (2017). Mature B cells accelerate wound healing after acute and chronic diabetic skin lesions. Wound Repair. Regen..

[40] Hassanshahi A., Moradzad M., Ghalamkari S., Fadaei M., Cowin A.J., Hassanshahi M. (2022). Macrophage-Mediated Inflammation in Skin Wound Healing. Cells.

[41] Chen R., Zou L. (2024). Combined analysis of single-cell sequencing and bulk transcriptome sequencing reveals new mechanisms for non-healing diabetic foot ulcers. PLoS ONE.

[42] Wu F., Wang J.Y., Chao W., Sims C., Kozar R.A. (2020). miR-19b targets pulmonary endothelial syndecan-1 following hemorrhagic shock. Sci. Rep..

[43] Li D., Peng H., Qu L., Sommar P., Wang A., Chu T., Li X., Bi X., Liu Q., Sérézal I.G. (2021). miR-19a/b and miR-20a Promote Wound Healing by Regulating the Inflammatory Response of Keratinocytes. J. Investig. Dermatol..

[44] Yang Z., Chen S., Ying H., Yao W. (2022). Targeting syndecan-1: New opportunities in cancer therapy. Am. J. Physiol. Cell Physiol..

